# Host Material Viscoelasticity Determines Wrinkling of Fungal Films

**DOI:** 10.1021/acsbiomaterials.4c01373

**Published:** 2024-09-24

**Authors:** Ciatta Wobill, Paride Azzari, Peter Fischer, Patrick A. Rühs

**Affiliations:** Institute of Food, Nutrition and Health, ETH Zürich, 8092 Zürich, Switzerland

**Keywords:** mycelium, wrinkling, soft material, engineered living materials, viscoelastic hydrogel

## Abstract

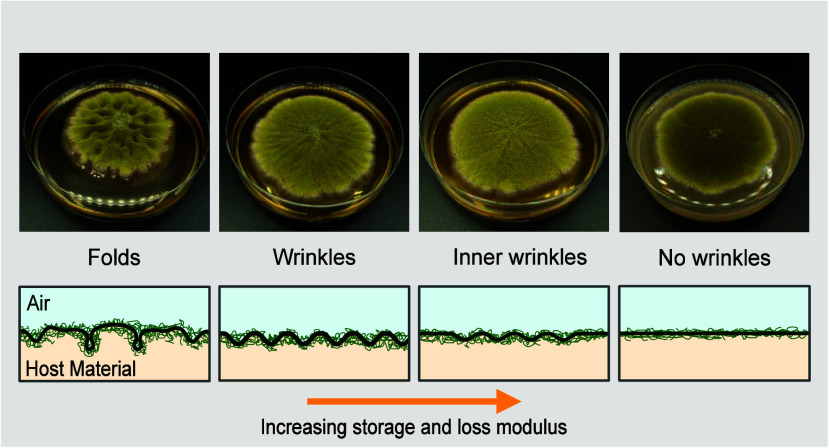

Microbial organisms react to their environment and are able to change it through biological and physical processes. For example, fungi exhibit various growth morphologies depending on their host material. Here, we show how the rheological properties of the host material influence the fungal wrinkling morphology. Rheological data of the host material was set in relation to the growth morphology. On host material with high storage modulus, the fungal film was flat, whereas on host material with low storage modulus, the fungus showed a morphology made of folds and wrinkles. We combined our findings with mechanical instability theories and found that the formation of wrinkles and folds is dependent on the storage modulus of the host material. The connection between the wrinkling morphology and the storage modulus of the host material is shown with simple scaling theories. The amplitude, number of wrinkles, and wrinkle length follow geometrical laws, and the mechanical properties of the fungal film are expected to increase with increasing host material elasticity. The obtained results show the connection between living biological films, how they react to their surroundings, and the underlying physical mechanisms. They can provide a framework to further design fungal materials with specific surface morphologies.

## Introduction

Soft biological material, such as lung alveoli, villi in the intestinal tract, and biofilms are prone to surface instabilities and are made of buckles and wrinkles. These structures allow nature to form complex and functional shapes, which can be observed at different length scales.^1^ At the molecular level, the confinement of molecules promotes the formation of bumps, which further leads to molecular gradients and induces biological responses.^2^ At a cellular and tissue level, cells grow nonuniformly and mechanical stress causes tissue deformation, such as in the intestine or lung alveoli. The curvature increases the surface area, leading to enhanced transport of nutrients and oxygen.^3−5^ The deformation can also be seen in plant and algae leaves. The green algae *Acetabularia acetabulum* forms spherical, leaf-like structures that change their wrinkling morphology as they grow.^6^ At the population level, the growth of bacteria in a colony leads to space confinement. To relieve the induced spatial stress, the biofilm wrinkles and buckles.^7^ This wrinkled morphology even leads to improved nutrient flow within the biofilm.^8^

To describe the mechanisms of wrinkling, various theoretical models and experimental data of sheets and films have been presented. In general, the sheets or films are on a layer of host material, making it a bilayer system. Mechanical instabilities in the system lead to wrinkling or folding to reduce the imposed spatial stress.^9,10^ For artificial systems such as polymer films or flexible electronics an external source such as stretching or indentation leads to wrinkle formation.^11,12^ In living biological films, stresses induced internally by the system during growth lead to a morphological change.^7,13,14^ Furthermore, delamination of the film from the host material can occur when the imposed stresses are too high. The wrinkling and delamination of both artificial and living films depend on the elastic rheological components of the film and host material, the thickness of the film, and the Poisson’s ratio. Often, the transgression of a critical stress then induces the morphological changes.^8,14−16^ Moreover, the substrate has a strong influence on wrinkling. For swelling-induced wrinkling the elasticity and height of this lower material lead to changing amplitude and wavelength of the film.^17^ Specifically for bacterial biofilms, the extent to which the host material influences the morphology is investigated. The free surface energy allows to control the delamination in *P. aeruginosa* biofilms,^8^ while changing the concentration of agar in the host material controls the wrinkling morphology of the biofilms.^18^

Although filamentous fungi in solid state fermentation have been gaining interest for example for the fabrication of leather alternatives, biomaterials, and food products, the effect of, and the adaptation to its host material, have been mostly neglected. Filamentous fungi are of particular interest because of their network structure formed of hyphae called mycelium.^19^ The fungal mycelium grows as an intricate structure to gather nutrients. The hyphae expand at the tip and branch apically or laterally to explore the surrounding host material.^20^ This growth occurs mainly at the edge of the culture.^21,22^ Meanwhile, it adapts its growth depending on pH, nutrients, and environmental conditions.^23−25^ The fungal structure is an ideal foundation to create so-called “engineered living materials”. Microorganisms are utilized to govern a response to environmental cues in a polymeric scaffold. This provides a novel approach for adaptable material.^26^ Especially, “smart living surfaces” that release functional molecules or respond to external cues are of interest.^27^ The adaptability, responsiveness to their environment, the structural integrity, and the production of highly surface active proteins of fungi provide a bandwidth of foundational properties useful for engineered living materials production.^27−30^

In this work, we demonstrate how the viscoelasticity of the host material influences fungal growth morphology and wrinkling. We investigate the driving forces for wrinkling and folding of the fungal film. The fungus was grown on various viscoelastic host materials, which were rheologically characterized. The experimental findings were complemented with a theoretical description of the wrinkling and compared to previous theories on wrinkling and mechanical instabilities. The study illustrates the relationship between living biological, and artificial film wrinkling and provides a framework that connects the underlying principles of these phenomena. The adaptation of fungi to their environment can be used to design engineered living materials and its host material with the prospect of creating sustainable, self-healing and growing materials. This research presents a foundation for smart living surfaces with tunable surface morphology.

## Materials and Methods

### Fungus, Growth Media, and Incubation

*Aspergillus oryzae* was selected as the model organism. We purchased a commercial spore powder (Kawashimaya Koji Starter for Shoyu, Hishiroku Shop Kyoto, Japan) and purified it to one phenotype over 5 incubation cycles. Then, the spores were harvested and used for further growth trials. A culture media composed of 2 wt % malt extract (Morga, Switzerland) and 0.2 wt % yeast extract (Sigma-Aldrich, France) was used. Additionally, polysaccharides agar (Morga, Switzerland), guar gum (Unipektin, Switzerland), or iota carrageenan (Danisco, Denmark) were added to vary the viscoelasticity. Inoculation was performed with the purified spores with a pipet tip into the middle of the host material and incubated for 7 days at 30 °C and a relative humidity of 90 to 95%.

### Confocal Microscopy and Image Analysis

Confocal laser scanning microscopy was used to analyze the surface profiles. The fungal films were prepared as mentioned in the previous section. After 7 days the surface of the cultures was scanned using a 5x objective on a confocal microscope (Zeiss Axiolmager.ZS, Carl Zeiss, Germany). Images were captured and converted into height maps using Zen Black 2012 (Carl Zeiss, Germany). Confomap (Carl Zeiss, Germany) was used to extract the depth of the wrinkles from the height maps. Additionally, pictures of the culture were taken with a standard camera (Nikon D800e, Nikon, Japan) equipped with a Nikon AF-S Nikkor 24–80 mm objective. Images were analyzed with FIJI (ImageJ). In brief, the number of wrinkles on the inner diameter and diameter of the culture as well as the wrinkling diameter were determined manually after fitting an appropriate threshold filter.

### Material Characterization

To investigate the rheological properties of the culture media, oscillatory shear tests were performed using a shear rheometer equipped with a plate plate geometry PP25, double gap geometry DG26.7, or Couette CC27 (MCR 702, Anton Paar, Austria). The temperature was kept constant at 30 °C with a Peltier bottom geometry. For amplitude sweeps a fixed frequency of 1 rad/s was used, while the strain increased stepwise from 1 to 100%. For frequency sweeps, the amplitude was fixed in the viscoelastic linear regime, while the frequency was increased stepwise from 0.1 to 100 rad/s. The values of storage and loss moduli for further analysis were chosen in the linear viscoelastic regime of the amplitude sweeps. Additionally, the viscosity of the host material was measured before and after fungal proliferation with a Couette CC27 geometry (MCR 702, Anton Paar, Austria). The density of media containing ι-carrageenan and guar gum was measured at 30 °C (DMA 500, Anton Paar, Austria).

## Results and Discussion

### Fungal Film Changes Morphology on Viscoelastic Host Media

**Figure 1:**
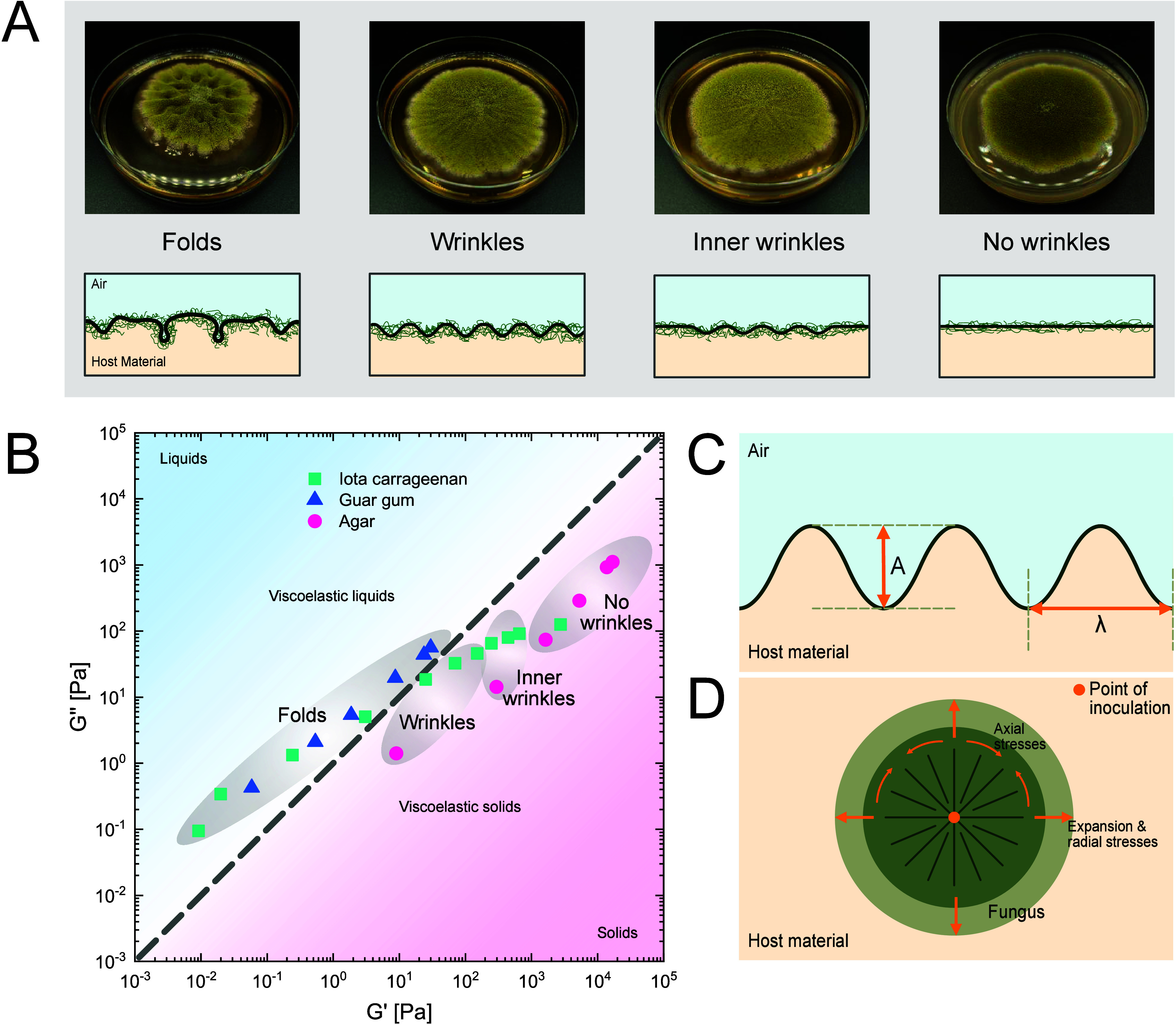
Overview of folding and wrinkling of fungal films. (A) Graphical overview of the four morphologies: with increasing storage and loss modulus the fungal morphologies change from folds to wrinkles, to inner wrinkles, and no wrinkles. Pictures were taken after 7 days of growth, and Petri dishes have a diameter of 9 cm. (B) Storage *G*′ and loss modulus *G*″ of host material and resulting fungal morphology. The dotted line represents a loss factor of 1. (C) Illustrative side view of wrinkled fungal films with the amplitude *A* and the wavelength λ. (D) Illustrative top view of the wrinkled fungal film: the inoculation point is in the middle, the fungus expands radially, and there are axial and radial stresses inducing the wrinkling.

After point inoculation and growth of the fungus on various growth media, we observed local wrinkling and folding of the fungal film. The fungus was grown in nutrient media adjusted with agar, ι-carrageenan, or guar gum. The rheological properties of these media were previously determined with oscillatory shear rheology. While growing in host material with increasing storage and loss modulus, the morphology followed a pattern. We identified four regimes of growth: fold-forming, wrinkle-forming, inner wrinkle, and flat regime. An overview of the four regimes and the corresponding moduli can be seen in Figure 1A and B. The wrinkling and folding can be decomposed in radial and angular waves^31^ and the largest curvatures of the fungal film were found closest to the inoculation point. At the point of inoculation, the mycelium is the oldest and experiences the highest amount of stress.^4,32^ In the fold-forming region, we observed both angular and radial waves with short wavelengths. These folds could even create holes in the fungal film, so the underlying host material was visible (Supplementary Image S1). In the wrinkle-forming regime, the folds turned into linear wrinkles, spreading radially to the growing front of the fungus. Here, angular waves with long wavelengths and radial waves with short wavelengths were formed. With increasing moduli, the inner wrinkle regime can be observed, which is a transition regime to the flat regime. In the inner wrinkle regime, we could still observe long angular waves, but the waves vanished before the growth front. The wrinkle length of the waves was smaller than the diameter of the fungal film. Lastly, the flat regime can be described with angular and radial waves of long wavelengths.

These four regimes followed the increase of the storage and loss moduli of the host material, where the transition between viscoelastic liquid and solid presented the transition region between fold and wrinkle forming regime. An overview of all media, their storage and loss modulus at the linear viscoelastic regime, and the resulting morphology can be seen in Figure 1B. The rheological measurements of the host material can be found in the Supporting Information (Supplementary Figures S2, S3, and S4). Viscoelastic liquids generally lead to fold formation, whereas viscoelastic solids lead to wrinkles or a flat morphology. The growth on guar gum always showed the hole-forming regime. With ι-carrageenan, we were able to observe the fold, wrinkle, and inner wrinkle-forming regime. Whereas for media containing agar, we were able to observe the wrinkling, inner wrinkling, and flat regime.

The system can be described with a bilayer model, where one layer—the fungus—expands radially on the compliant second layer—the host material. The compliant host material refers to a material which can be shaped by the film that grows on top.^33,34^ Upon expansion of the fungus, in-plane stresses arise and these lead to folding or wrinkling depending on the viscoelastic properties of the host material. An illustration of the fungal growth on the host material can be found in Figure 1C and D, including the stresses acting in the fungal film during growth.

In conclusion, fungal film wrinkling is unique in the realm of wrinkling instabilities in the literature, especially in living biological materials. The fungal biofilm is neither a system on cellular level nor a system on population level. Unlike bacterial biofilms at the population level, fungal films do not delaminate due to their growth mechanism. Fungal proliferation is fundamentally different from bacterial biofilm growth: While bacteria are unicellular organisms forming biofilms, most of which do not penetrate solid surfaces,^35^ filamentous fungi are multicellular and form a densely packed network from individual hyphae.^19^ This network penetrates the host material, which should prevent delamination. At the cellular level, the fungal biofilms behave comparable to the intestinal wall.^4^ Connected cells expand and space confinement leads to out-of-plane wrinkling. Similar morphologies to fungal film wrinkling are found in floating sheets that are indented to induce stress.^31,36,37^ There, wrinkling occurs after azimuthal compression and results in radial wrinkles. Overall, the morphology of wrinkling fungal biofilms resembles bacterial biofilms and artificial film wrinkling found in literature.

### Viscoelastic Material Properties Control Wrinkling of Fungal Films: A Bilayer Model Can Predict the Transition from Wrinkled to Flat Fungal Films

In the following section, we propose possible mechanisms for the wrinkling of fungal films supported with an appropriate model to rationalize our experimental findings. First, the transition from flat to wrinkled will be discussed.

We hypothesize that the main reasons for wrinkling are mechanical instabilities that arise during the radial growth of the fungal film. In traditional mechanical instability theories, to reduce the induced stress,^16^ the film either wrinkles as for plastic films^11,38^ or delaminates after wrinkling from the underlying material as found for bacterial biofilms on solid surfaces.^8^

We describe our system as a bilayer of fungus bonded to the compliant host material. The fungus expands on the host material, and in-plane compressive forces of the fungus in radial and axial directions lead to a folding or wrinkling of the fungal film, without delamination. An explanatory illustration of the fungal film on the host material and the resulting stresses can be found in Figures 1C and D, respectively. We adapt a theory by Huang et al.^34^ The model introduces the energy of a wrinkling system as a fourth order polynomial of the amplitude *A*. The total energy stored in the film *U*_*total*_ can be described as follows in eq 1:
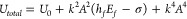
1The wrinkles have an amplitude *A* and wavelength λ; the fungal film thickness is *h*_*f*_; the elastic modulus of the film is *E*_*f*_; the critical stress is σ; the inner diameter is *R*; and *k* = (*Rπ*)/λ. Hereby, the film is in a local energy minimum if *h*_*f*_*E*_*f*_ – σ > 0, *A* = 0, and the film is flat. If *h*_*f*_*E*_*f*_ – σ < 0 and *A* is in equilibrium, the film is at a local energy maximum and wrinkles. Here the stresses in the system exceed a critical stress as shown in eq 2:
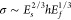
2The critical stress of the system depends on the thickness *h*_*f*_ of the film, the elastic modulus of the film *E*_*f*_ and of the substrate *E*_*s*_. The main parameter, which determined whether the fungal film wrinkled, was the elastic material property *E*_*s*_. We approximate the storage modulus *G*′ of the host material, measured with oscillatory shear rheology, as the elastic modulus *E*_*s*_. Using eq 2, we can state if *E*_*s*_^2/3^ ∼ *G*′ > σ/(*h*_*f*_*E*_*f*_^1/3^) the system is flat, if *E*_*s*_^2/3^ ∼ *G*′ < σ/(*h*_*f*_*E*_*f*_^1/3^) the system is wrinkled or folded. Our system wrinkles or folds at *G*′ up to about 658 Pa, i.e., the host material is soft enough to be deformed by the fungus. Exceeding this value, the films become flat. Thus, we can state, that with increasing storage modulus of the host material, the fungus has less ability to deform the material and does not wrinkle anymore. Although we cannot state an exact number for the critical stress at which the fungal film wrinkles, we can control the wrinkling status simply by defining the elastic modulus.

There have been no studies on the wrinkling of fungal films. Previous studies have identified critical stresses for wrinkling, buckling, and delamination of thin films,^11^ electronics^38^ or for bacterial biofilms.^8,14,39^ Most driving factors are similar, e.g., substrate elasticity, film thickness, and film elasticity. Nevertheless, the systems are only comparable to an extent. A majority of the beforementioned literature discusses films that wrinkle with increasing stress.^14,18,38^ Our system wrinkles at low stresses, where the host material is soft enough to be deformed. Above the critical stress, the system becomes flat due to the increase in modulus of the substrate.

High elasticity prevents the fungus from deforming the surface, at low elasticity the fungus can overrule the pressure of elasticity to flatten the surface and create a wrinkled surface. Similar behavior can be found in nature. Hannezo et al.^4^ discuss that the dimension of villi in the intestinal tracks depends mainly on the elastic parameters of the cell layers. Two examples from Huang et al.^15^ and Huang et al.^34^ illustrate the extent to which substrate rheology influences wrinkle morphology. They investigated differences between purely elastic and viscous or viscoelastic substrates and to which extent the critical stresses are affected. Unlike elastic or viscous substrates, viscoelastic substrates affect wrinkling severely, and the wavelength and amplitude are directly impacted by the substrate moduli.

### Bilayer Model Can Predict Geometrical Parameters of the Wrinkles

**Figure 2:**
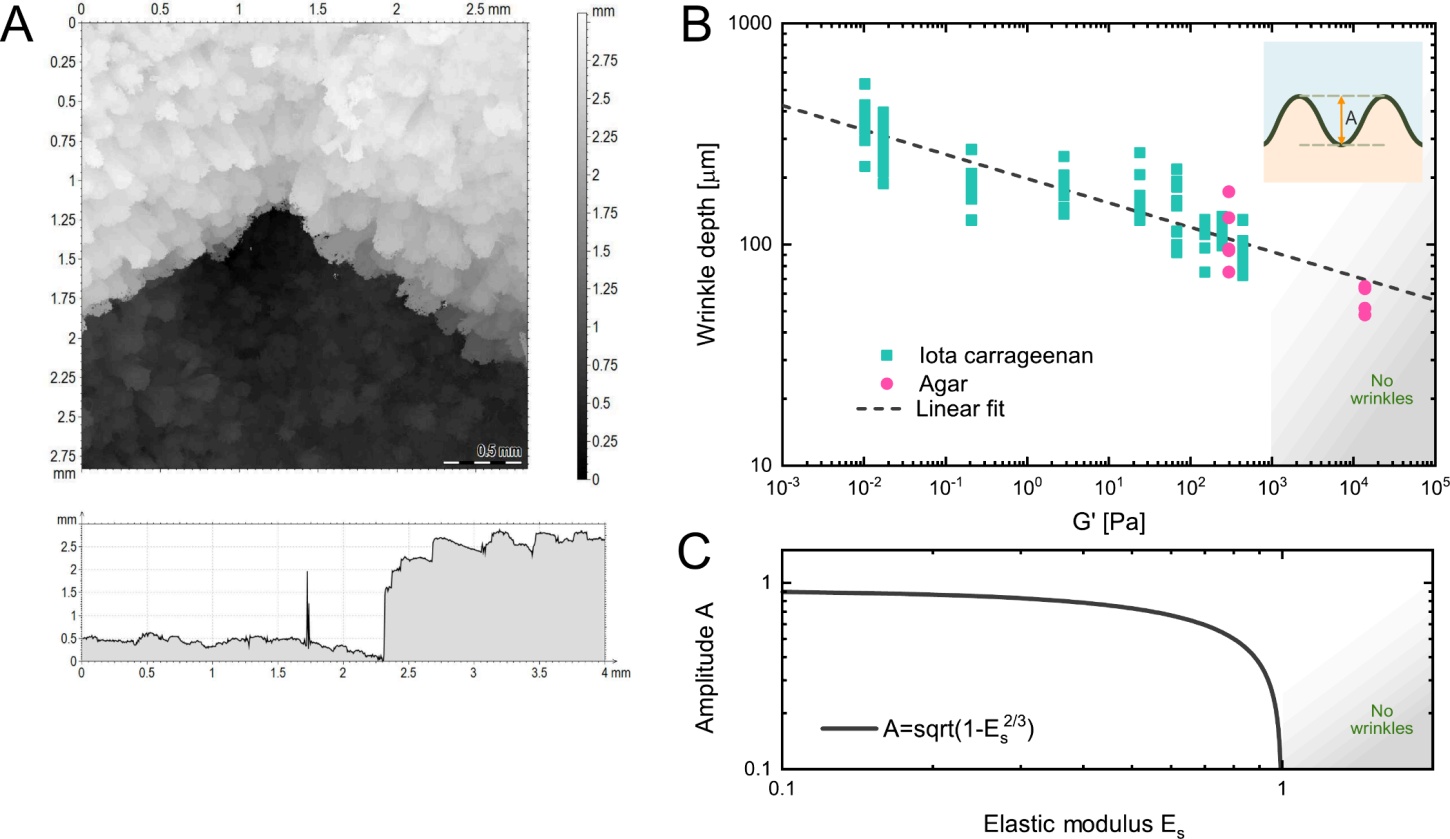
Dependance of the wrinkle amplitude on the storage modulus of the host material. (A) Exemplary measurement from film grown on 0.5 wt % ι-carrageenan. Measurements were taken after 7 days of growth. (B) Depth of wrinkles and folds measured with confocal microscopy plotted against the storage modulus *G*′ from the respective host materials. The linear fit led to a slope of −0.11 ± 0.01 and an *R*^2^-value of 0.81. Above 657.95 Pa the fungal films were flat. (C) Plot of eq 3 for varying *E*_*s*_ with all other parameters held constant. Above a certain elastic modulus, here 1, the films become flat.

Next, we investigated the patterns of the fungal films given by the amplitude, the number of wrinkles, and the inner radius, which were related to eq 1. To extract the amplitudes of the films, we measured the depths of multiple wrinkles and folds using confocal scanning microscopy. Exemplary data from a film grown on 0.5 wt % ι-carrageenan can be found in Figure 2A. The corresponding data of all films are plotted in Figure 2B as a function of the storage modulus of the respective host material. With the storage modulus increasing, the depth of the wrinkles and folds decreases. This was measured for all films grown in media containing ι-carrageenan and for two media containing agar. The lowest value measured was for the material with the highest elastic modulus, 2 wt % agar. Here the film was flat, which means that a constant height of 48.1 μm with the additional height of aerial mycelium and the conidiophores was measured.^40,41^ In addition, the data were fitted with a linear regression, resulting in a slope of −0.11 ± 0.01. Therefore, the amplitude scales the elastic modulus *E*_*s*_ with an exponent of −1/9 with an *R*^2^-value of 0.81.

Assuming the system follows eq 1, the amplitude can be approximated as follows in eq 3:
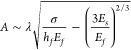
3with the wavelength in eq 4:
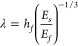
4The amplitude depends on the wavelength λ, the critical stress σ, the height *h*_*f*_, and elastic modulus of the film *E*_*f*_, as well as the elastic modulus of the host material *E*_*s*_. The wavelength is calculated with the rheological properties of host material and fungal film. We can substantiate our data with eq 3: The wrinkle amplitude and wavelength decrease with increasing elastic properties of the substrate. To visualize the implications of the eq 3, one can simplify eq 3 to depend only on the elastic modulus *E*_*s*_ and get . The amplitude *A* as a function of only *E*_*s*_ is shown in Figure 2 C. Above a certain elastic modulus, here *E*_*s*_ equals 1, the biofilms become flat and the amplitude is zero.

**Figure 3:**
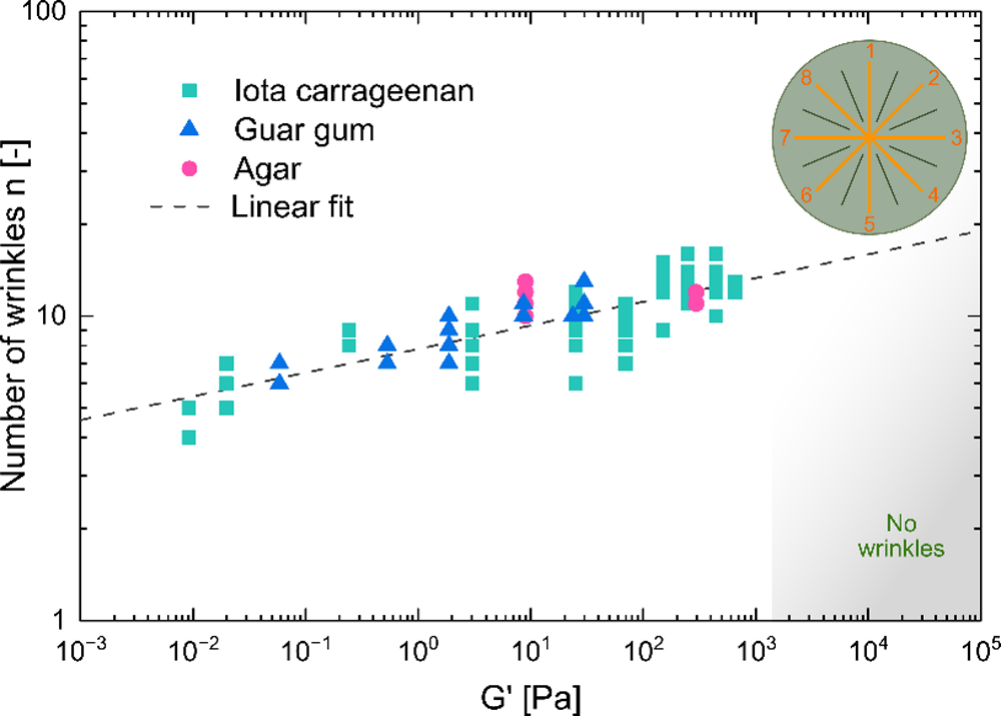
Relationship between the number of wrinkles *n* and the storage modulus *G*′ of the host material of the host material. The linear fit has a slope of 0.08 ± 0.01 with an *R*^2^-value of 0.69.

We also measured the number of wrinkles and folds radially closest to the point of inoculation. These are the oldest wrinkles and folds and had the most time to develop. Figure 3 shows the interaction between the number of wrinkles *n* and the storage modulus *G*′. As the storage modulus increases, so does the number of wrinkles. We conducted measurements across all media in which the films were not flat. A linear fit was applied, yielding a slope of 0.08 ± 0.01 with an *R*^2^-value of 0.69 for the fit.

**Figure 4:**
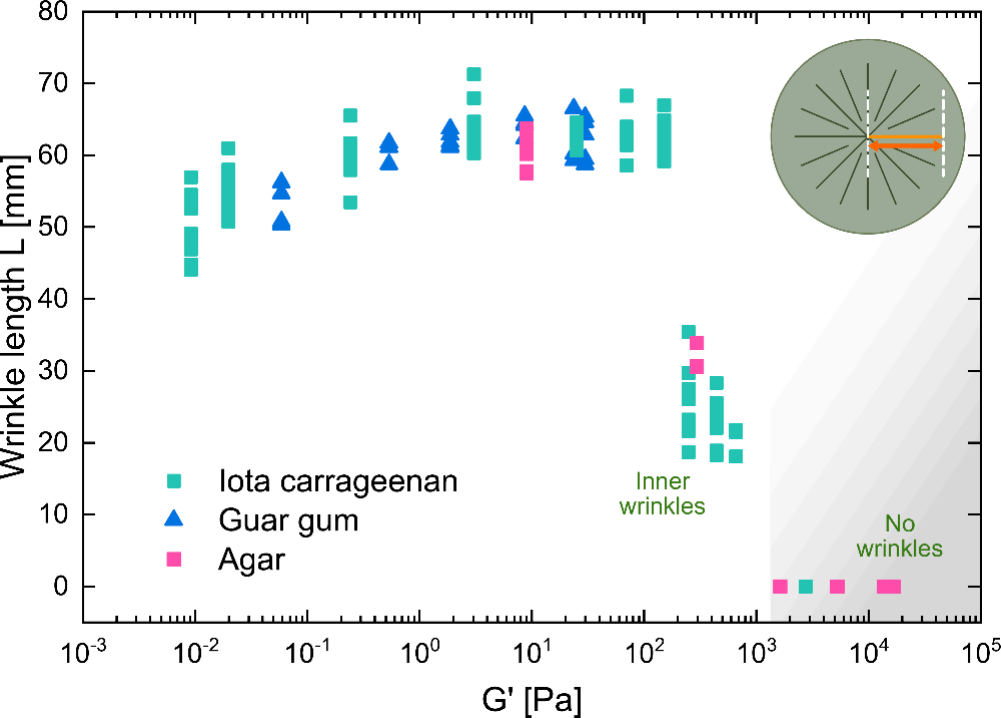
Relationship between the wrinkle length *L* and the storage modulus *G*′ of the host material.

The model of Chen and Hutchinston^42^ predicts *n* = 2*πR*/λ, which we can approximate to the following eq 5 using eqs 1 and 3:
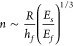
5Additionally, we looked at the wrinkle length *L* of a wrinkle, which is the separating factor between the wrinkle regime and the inner wrinkle regime. In the wrinkle regime, there were long angular waves that spanned over the entire fungal film, meaning the wrinkle length was equal to the radius of the culture. In contrast, within the inner wrinkle regime, the wrinkle length was smaller than the radius of the culture. We found an increase in wrinkle length from the fold to the wrinkle regime, followed by a plateau and an immediate drop to the inner wrinkle regime as shown in Figure 4.

Using arguments from earlier work in uniaxial geometries extended to a radial geometry,^43^ the length for the fungal films can be approximated as follows in eq 6, with the wavelength λ, the critical stress σ, and the film thickness *h_f_*:
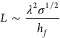
6The values found for the scaling laws of the amplitude and number of wrinkles have a geometrical origin.^34,42^ Reducing the amplitude results in an increase in the number of wrinkles, coupled with a corresponding decrease in the wavelength. Similar results were found in the literature for thin films and bacterial biofilms for all measured values.^18,37^

Thin film wrinkling due to indentation has shown to be most comparable. The amplitude, number of wrinkles and wrinkle length in various studies showed similar trends and relations to the host material. The energy required to deform the substrate increased as more energy was added to the system, either by indentation or by weight. Nikravesh et al.^33^ simulated the size of the amplitude of thin films and found a dependence on the energy added to the system and the same behavior with , just dependent on the applied strain γ rather than the storage modulus. Holmes et al.^36^ found an increase in the number of wrinkles as a function of the thickness and elasticity of the film. The wrinkle length increased when adding more stress.^15,31,37,44^

To conclude, the amplitude and number of wrinkles are as expected for a film, with respect to a radial geometry. The wrinkle length can be used to identify the transition from the wrinkle to the inner wrinkle regime. Due to the fungal network formation and penetration into the material, the wrinkling morphology differed from other materials.

### Increase in Storage and Loss Moduli Limits the Bendability of the Fungal Film and Host Material: Transition from Folds to Wrinkle Regime

Previously described transitions, from flat to wrinkled and inner wrinkled to wrinkled, followed the increase of the storage modulus. However, an increase in storage modulus cannot easily explain the transition between the folds and the wrinkle regime. Similar results were seen by Hannezo et al.^4^ and by Huang et al.^45^ They saw that the structure of the intestinal wall in their simulation was dependent on the pressure or stresses applied. After a boundary increase, the membrane was no longer able to bend in all directions, changing the morphology. Therefore, we assume that at low storage and loss modulus, the fungus can deform the host material in both angular and radial directions which results in the fold structure. However, with increasing moduli, it is not possible to bend the host material in both radial and angular directions. Here, only angular waves can be formed. The radial waves have such a long wavelength, they do not present in the morphology and only waves can be observed.

### Scaling Relationship between Film Wrinkling Wavelength and Film Elasticity

In the following, we present predictions for the wavelength λ and the elastic modulus of the fungal film *E*_*f*_. With scaling laws found for the amplitude *A* and the number of wrinkles *n*, we can hypothesize on the scaling laws between the wavelength λ and the elastic modulus of the host material *E*_*s*_. Further, a scaling law between elastic modulus of the fungal film *E*_*f*_ and the elastic modulus of the host material *E*_*s*_ can be calculated. However, some assumptions need to be discussed first. Although we have not measured the fungal film thickness *h*_*f*_, it is assumed that the thickness decreases with increasing storage and loss modulus. Literature on fungal growth on solid substrates illustrate that a decrease in penetration depth is associated with an increase in material density,^46−49^ mostly due to the lower oxygen concentration.^50^ We measured an increased density for a higher concentration of thickening agents (Supplementary Figure S5), which would indicate a negative exponent for the scaling law of the film thickness *h*_*f*_ and the elastic modulus of the substrate: *h*_*f*_ ∼ *E*_*s*_^–α^, where α > 0.

Additionally, we measured the wrinkling radius of all films grown on ι-carrageenan. The wrinkling radius decreased with increasing storage modulus with a slope of −0.13 ± 0.01 and an *R*^2^-value of 0.813 (Supplementary Figure S6). Using eqs 3 and 5 we can calculate the scaling law between the wavelength λ and the elastic modulus of the substrate *E*_*s*_: λ ∼ *E*_*s*_^–0.05^. Including our assumption for the fungal film thickness *h*_*f*_, the elastic modulus of the fungal film *E*_*f*_ scales as follows in eq 7:
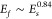
7More detailed calculations for eq 7 can be found in the Supporting Information. We can predict that the elasticity of the fungal film would increase as the elasticity of the host material increases. While the elasticity of fungal films has been measured in previous studies,^25,51^ the effect of the host material viscoelasticity has not been considered. Hotz et al.^52^ looked at the influence of the agar concentration in the substrate and reported an increase in mycelium density for higher agar concentrations. Nussbaum et al.^25^ found that at higher mycelium density the elastic modulus of the film increased. Combining these findings, we can conclude that an increasing density would lead to a higher elastic modulus, thereby confirming our approximations. Further studies could include the measurement of the fungal film elasticity by tensile tests.

## Conclusion

**Figure 5:**
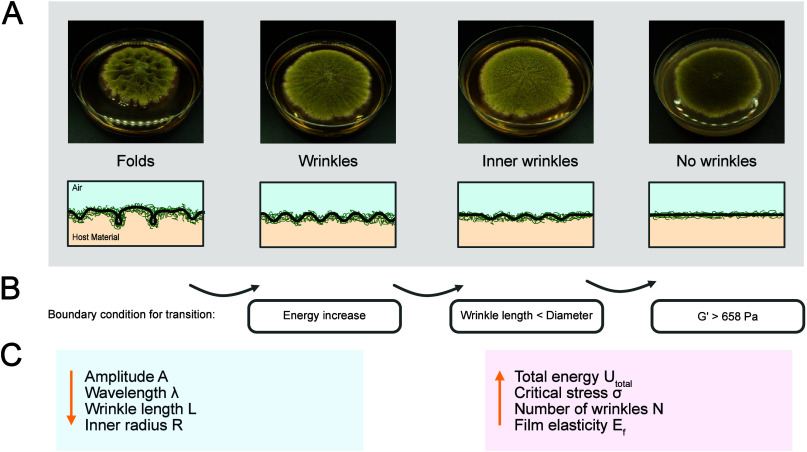
Overview of folding and wrinkling of fungal films. (A) Graphical overview of the four morphologies: with increasing storage and loss modulus the fungal morphologies change from folds to wrinkles, to inner wrinkles and no wrinkles. Pictures were taken after 7 days of growth, and Petri dishes have a diameter of 9 cm. (B) The boundary conditions for the transitions are the following: an overall energy increase leads to the transition from the folds to the wrinkle regime. The decrease in wrinkle length, from the wrinkle length being equal to the radius of the culture to being smaller than, equates to the transition from wrinkle to inner wrinkle regime. Above a storage modulus value of 658 Pa of the host material, the fungal films become flat. (C) With increasing storage and loss modulus the amplitude, wavelength, wrinkle length, and inner radius decrease, while the total energy, critical stress, number of wrinkles, and the film elasticity increase.

We studied the film wrinkling of fungal films and related it to the storage and loss modulus of the host material. An overview of the results can be found in Figure 5. To describe the morphology, four different regimes were categorized and successfully approximated by theoretical models. Figure 5 A shows an overview of the four regimes, Figure 5 B shows the boundary conditions for transition, and Figure 5 C the implications of changing the host material on the parameters. In short, the transition between flat and wrinkled depends on the elastic modulus. When the critical stress is exceeded, the fungal film becomes flat. When the film wrinkles, we can describe the amplitude, number of wrinkles and wrinkle length with appropriate equations that follow from the total energy in the system. The transition between the inner wrinkle and the wrinkle regime could be explained by an equation describing the length of the wrinkles. The transition from the folds to the wrinkle regime does not directly follow the increase of the storage modulus of the host material but seems to be a bendability issue when increasing the stress in the system. Lastly, the elasticity of the film is predicted to increase with the storage modulus of the substrate. The fungal film wrinkling phenomena show similarities to other systems previously described in nature, such as the intestinal wall and bacterial biofilms, as well as thin film wrinkling. The results demonstrate the importance of the rheological properties of the host material in controlling fungal film growth. Further studies could explore the effective elastic properties of the fungal film using dynamic mechanical analysis or dilatational rheology. However, proper material collection and handling would be crucial. We were able to show that this material aspect influences the fungal morphology. Thus, we can control fungal morphology and mechanical properties simply by changing the viscoelasticity of the host material. This result can be used to specifically design host material for fungal proliferation and smart living surfaces with a desired surface morpholgy.

## Supporting Information Available

Additional calculation details, image of a fungal culture in Figure S1, rheological measurements in Figures S2–S4, density measurements in Figure S5, and the wrinkling radius measurements in Figure S6 (PDF)
