# Supplementary material for: Host Material Viscoelasticity Determines Wrinkling of Fungal Films
Source: ACS Biomater Sci Eng. 2024 Sep 24;10(10):6241–9. doi: 10.1021/acsbiomaterials.4c01373 (PMC11480942; doi:10.1021/acsbiomaterials.4c01373)
Supplement: Supplementary file 1 — ab4c01373_si_001.pdf [file ab4c01373_si_001.pdf]

Supporting Information: Host material viscoelasticity determines wrinkling of fungal films

Ciatta Wobill, Paride Azzari, Peter Fischer, and Patrick Rühs*

Institute of Food, Nutrition and Health, ETH Zürich, 8092 Zürich, Switzerland

E-mail: patrick.ruehs@hest.ethz.ch

Phone: +41 44 632 36 68

Scaling relationship between film wrinkling wavelength and film elasticity - Detailed calculations

In this section we outline the relationship between the film wrinkling wavelength λ, the elasticity of the film E_f and of the substrate E_s, which are obtained from the storage modulus G' introduced in the main text.

From measuring the wrinkling radius R for different samples, the amplitude of the wrinkles A and the number of wrinkles n around the radius we find the following numerical relationships:

$$R \sim E_s^{-0.13} \tag{1}$$

$$A \sim E_s^{-0.11} \tag{2}$$

$$n \sim E_s^{-0.078} \tag{3}$$

where $n = \frac{2\pi R}{\lambda}$.

From a scaling argument we obtain

$$\lambda \sim \frac{R}{n} \sim \frac{E_s^{-0.13}}{E_s^{0.078}} = E_s^{-0.05}, \tag{4}$$

therefore

$$\lambda = E_s^{-0.05}. \tag{5}$$

From Huang *et al.*[1] we can estimate λ as

$$\lambda \sim h_f \left(\frac{E_f}{E_s}\right)^{1/3} \sim E_s^{-0.05} \tag{6}$$

which can be simplified to derive

$$h_f E_f^{1/3} = E_s^{0.28} \tag{7}$$

In the hypothesis where the height of the film h decreases for higher substrate elasticity we can define a $\alpha > 0$ such that

$$h_f \sim E_s^{-\alpha}, \tag{8}$$

Moreover, if $E_f \sim E_s^{\beta}$, we get

$$-\alpha + \frac{\beta}{3} = 0.28, \tag{9}$$

That reduces to

$$\beta > 0.84 \quad \text{if} \quad \alpha > 0 \tag{10}$$

If the thickness of the film decreases for higher elasticities then the elasticity of the film must grow according to the following power law:

$$E_f \sim E_s^{0.84}. \tag{11}$$

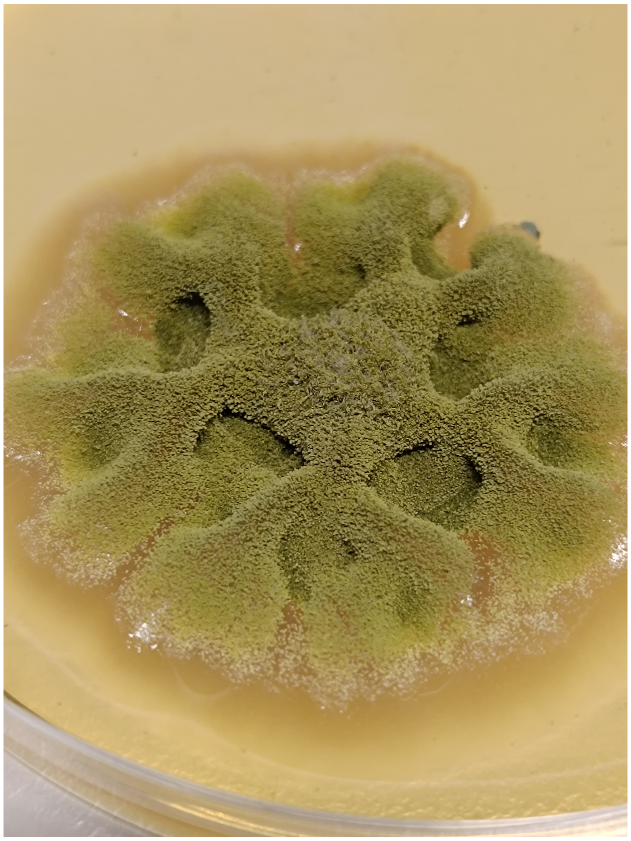

Figure S1: Image of culture grown on guar gum containing media: The fungal film formed holes and the underlying material is visible.

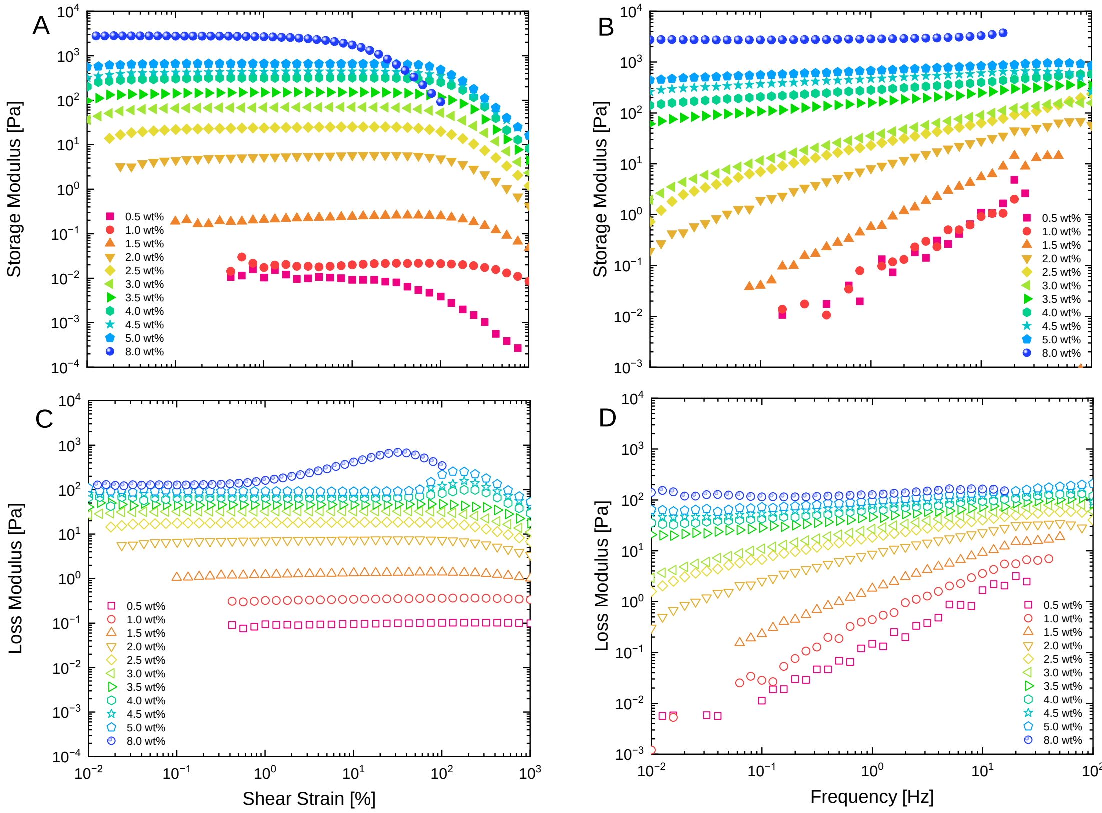

Figure S2: Figure S2 shows the rheological characterisation of all iota carrageenan containing media: Figures A and C show the storage modulus and loss modulus of the amplitude sweeps and figures B and D the storage and loss modulus of the frequency sweeps.

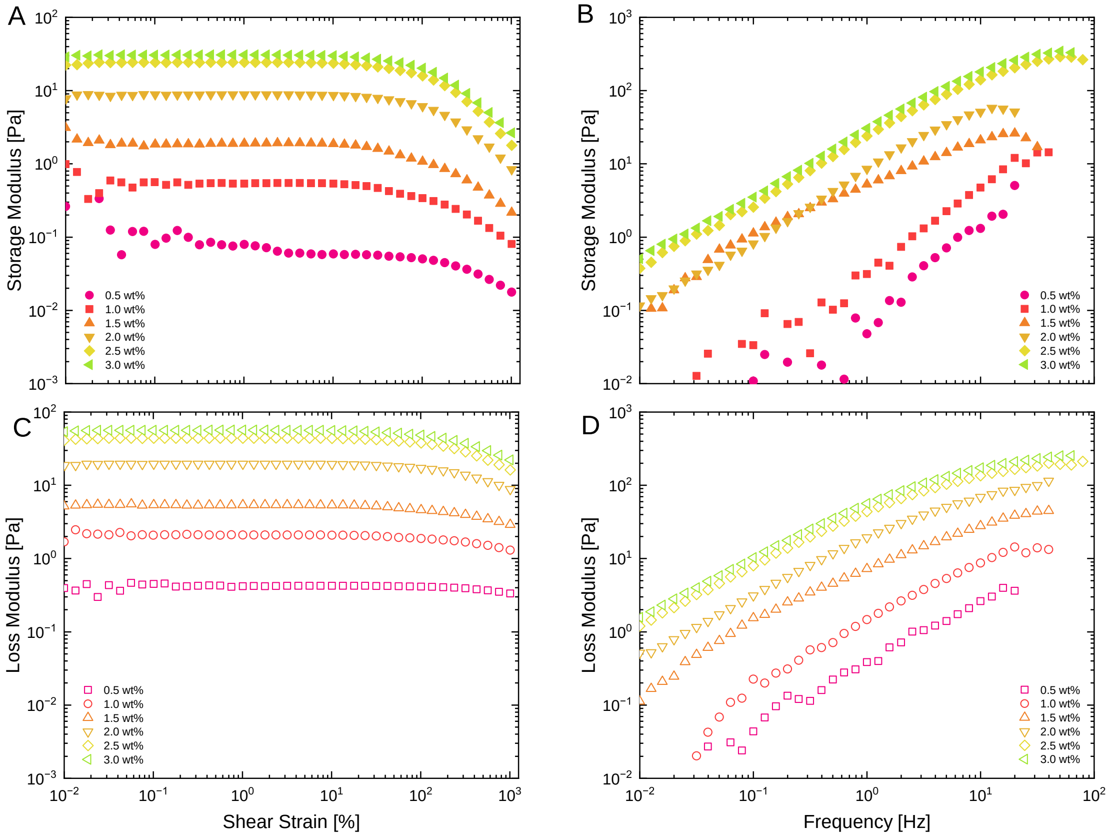

Figure S3: Figure S3 shows the rheological characterisation of all guar gum containing media: Figures A and C show the storage modulus and loss modulus of the amplitude sweeps and figures B and D the storage and loss modulus of the frequency sweeps.

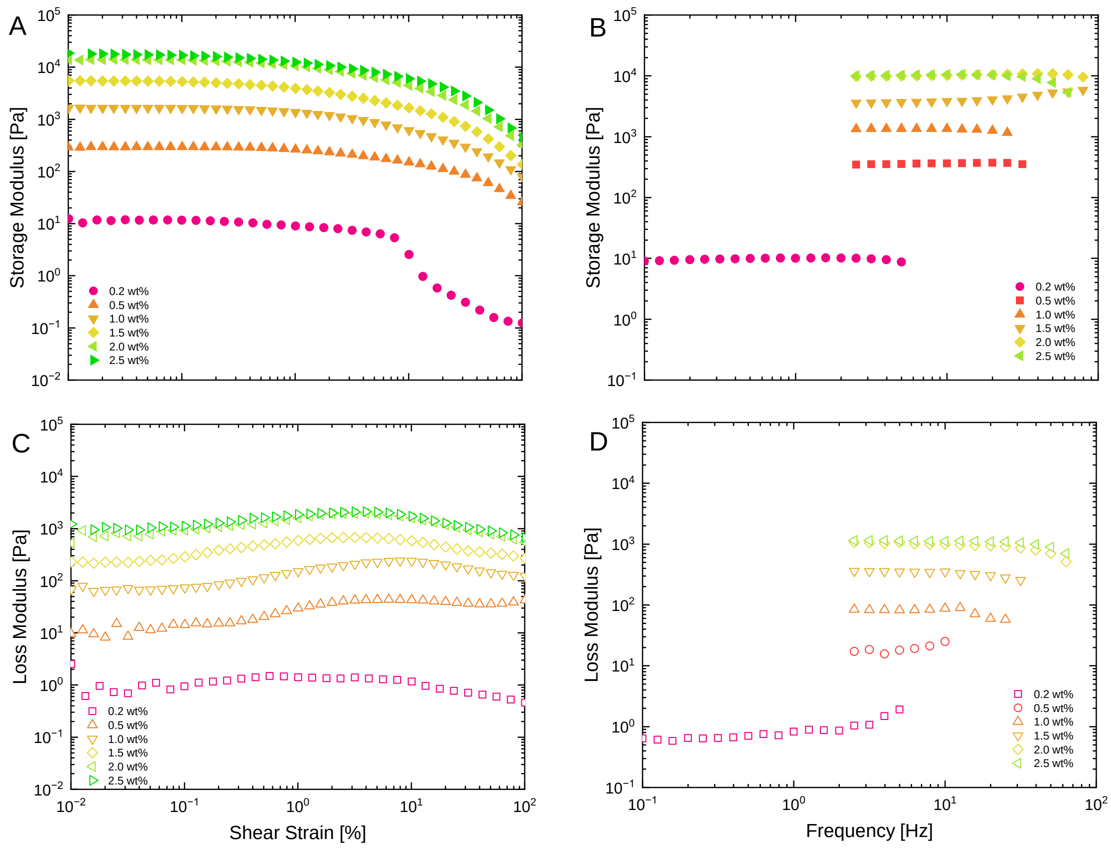

Figure S4: Figure S4 shows the rheological characterisation of all agar containing media: Figures A and C show the storage modulus and loss modulus of the amplitude sweeps and figures B and D the storage and loss modulus of the frequency sweeps.

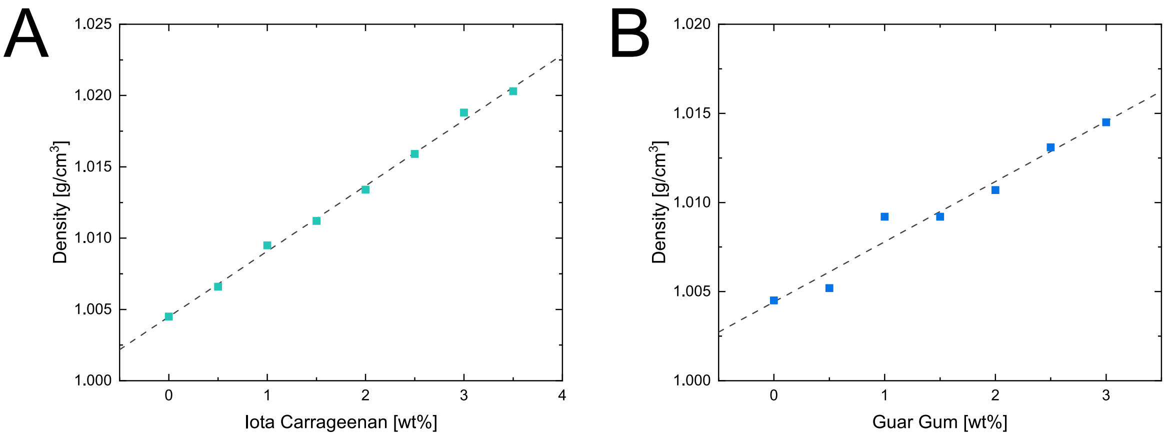

Figure S5: Density of iota carrageenan and guar gum media with increasing concentration of hydrogels: Figure A shows the densities for media containing iota carrageenan. The linear fit has a slope of 0.01 ± 1.03 and an R^2 of 0.997. Figure B shows the densities for media containing guar gum. The linear fit has a slope of 0.01 ± 3.01 and an R^2 of 0.962.

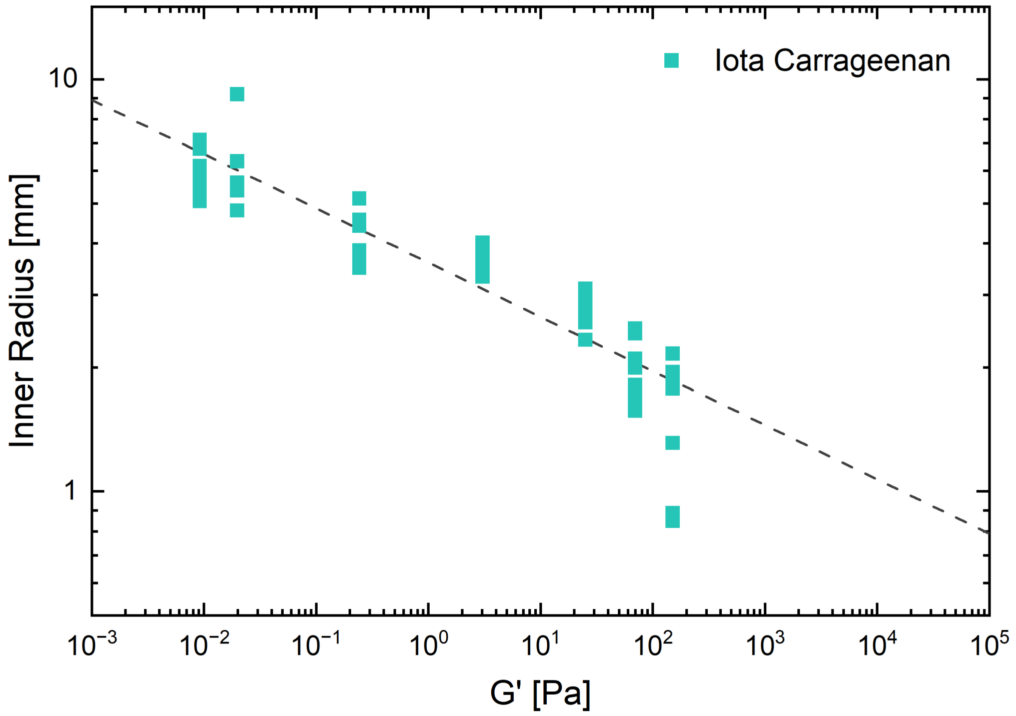

Figure S6: The wrinkling radius decreased with increasing storage modulus with a slope of -0.13 ± 0.01 and an R^2-value of 0.813.

References

(1) Huang, Z.; Hong, W.; Suo, Z. Nonlinear analyses of wrinkles in a film bonded to a compliant substrate. *Journal of the Mechanics and Physics of Solids* **2005**, *53*, 2101–2118.
